# Supplementary material for: A Multivariant Surrogate Neutralization Assay Identifies Variant-Specific Neutralizing Antibody Profiles in Primary SARS-CoV-2 Omicron Infection
Source: Diagnostics (Basel). 2023 Jul 5;13(13):2278. doi: 10.3390/diagnostics13132278 (PMC10341213; doi:10.3390/diagnostics13132278)
Supplement: Supplementary file 1 [file diagnostics-13-02278-s001.zip › diagnostics-2474055-supplementary.pdf]

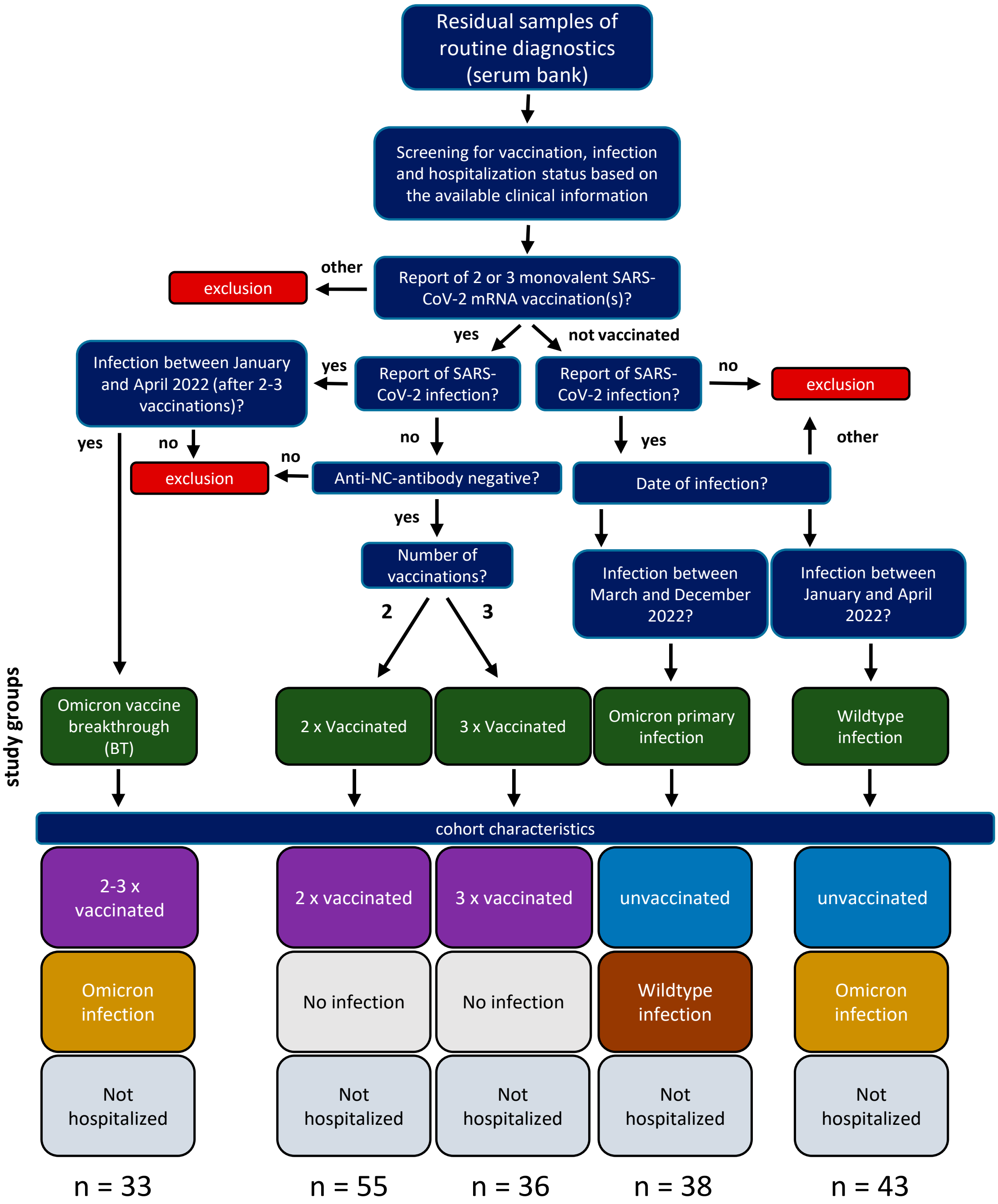

Figure S1. Flow chart depicting the inclusion process of the serum samples as well as an overview over general cohort characteristics.

Note that some differences (e.g. time after immunization) were accepted due to the availability of the sera, since the rationale was to include sera that cover a wide range of titer levels as well as sera from Omicron primary infected subjects.

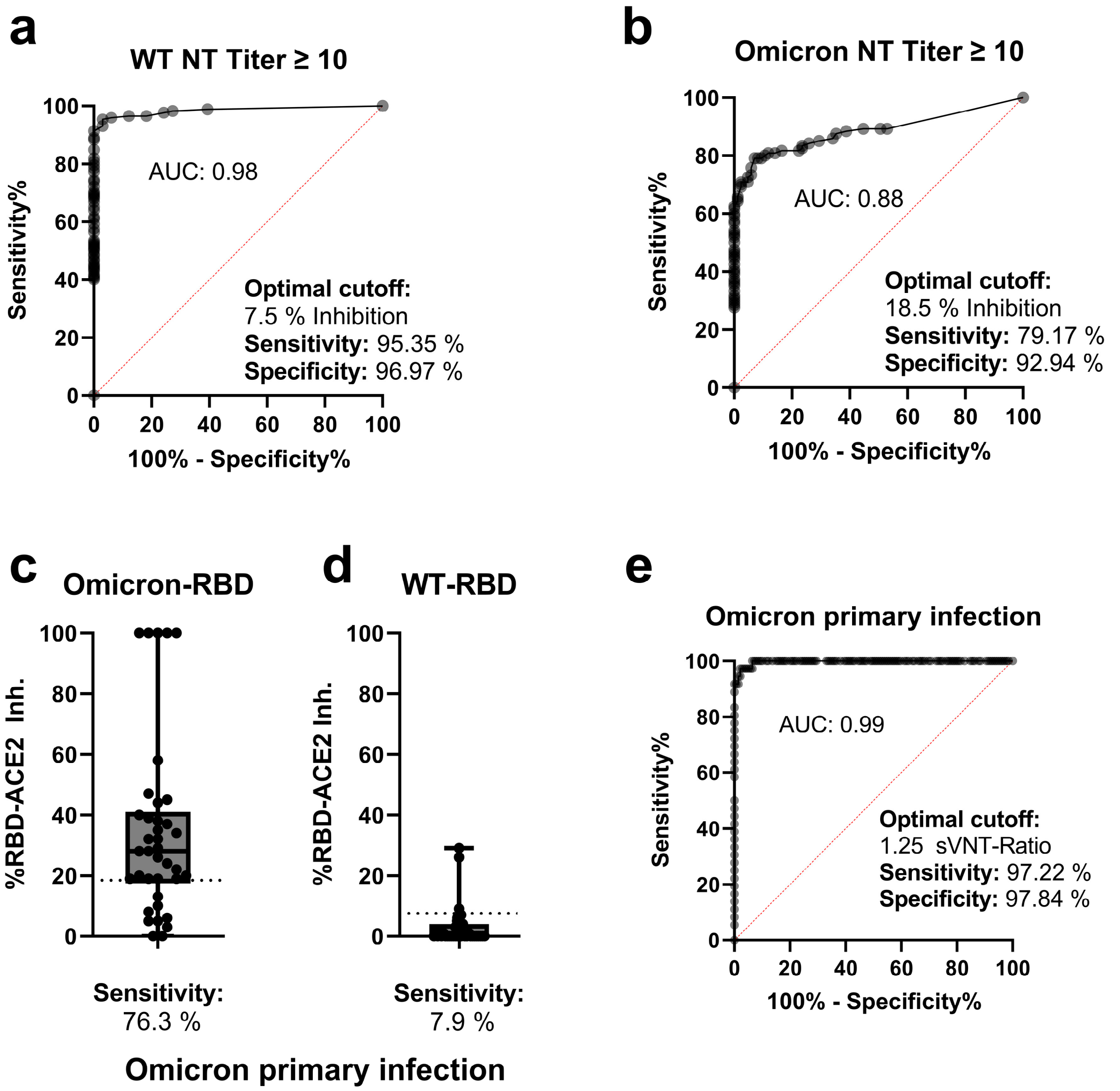

Figure S2. Sensitivity and specificity of the sVNT to determine a NT titer ≥ 10 or to detect Omicron primary infection.

a, b) ROC analysis to determine **a)** the optimal WT-RBD-ACE2 inhibition threshold that reflects a WT NT titer ≥ 10 and **(b)** the optimal Omicron-RBD-ACE2 inhibition threshold that reflects a Omicron NT titer of ≥ 10. **c, d)** Sensitivity of the **(c)** sVNT Omicron-RBD-ACE2 inhibition and **(d)** sVNT WT-RBD-ACE2 inhibition results to detect neutralizing activity in the Omicron primary infected cohort applying the thresholds determined in **(a)** and **(b)** (n = 38, note that all samples had an Omicron NT titer ≥ 10). **d)** ROC analysis to determine the optimal sVNT-Ratio threshold to distinguish the Omicron primary infected cohort from the other cohorts. AUC: area under the curve.

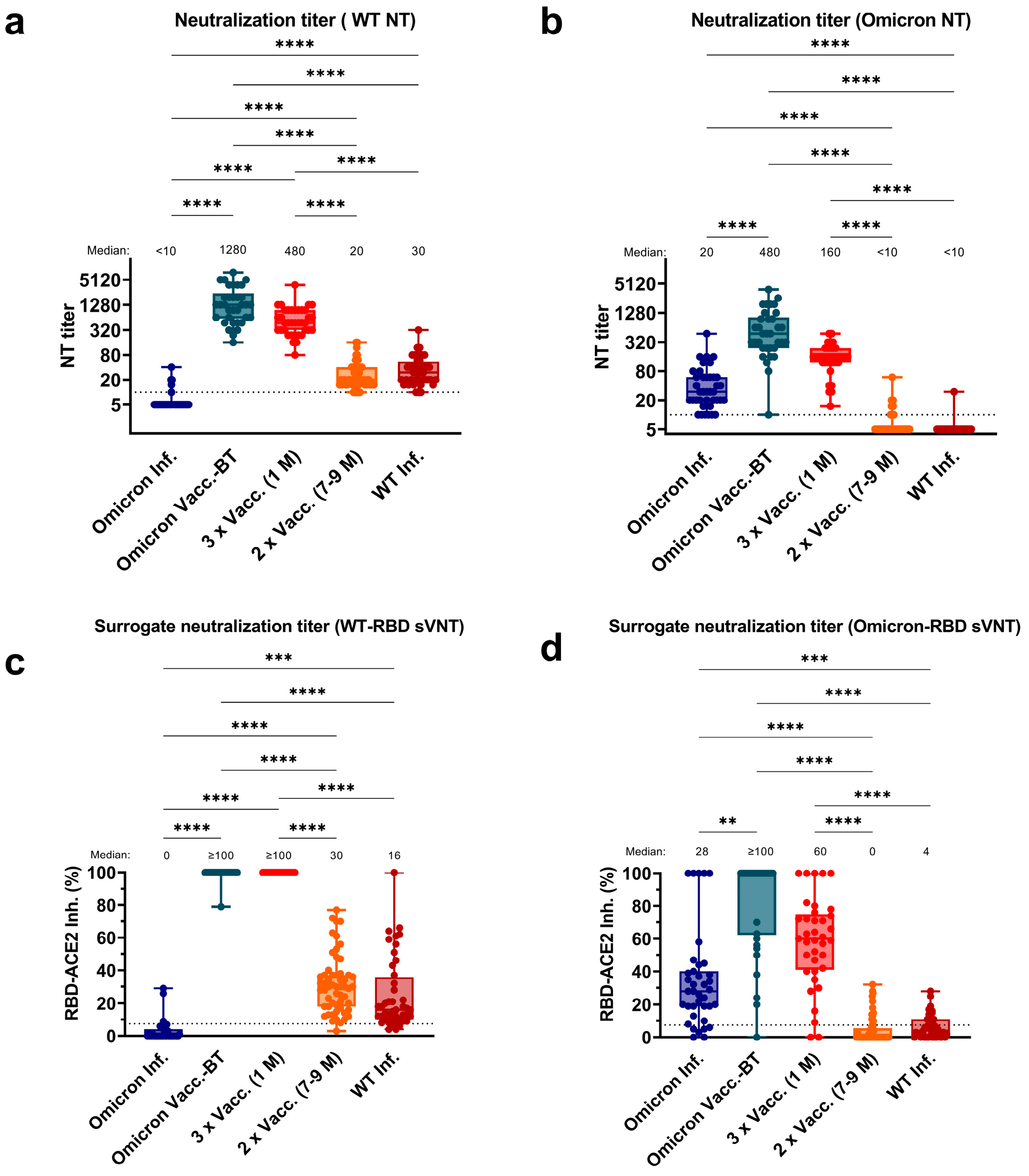

Figure S3. Cohort-wise comparison of neutralizing activity using the live-virus NT as well as the sVNT.

a, b) Comparison of **a)** WT-NT titer and **b)** Omicron-BA.1 NT titer between the cohorts. **c, d)** Comparison of **c)** WT-RBD-ACE2 Inhibition and **d)** Omicron-RBD-ACE2 Inhibition (%). A Kruskal-Wallis test was significant for all four panels; shown are significance levels of multiplicity-adjusted Dunn's post-hoc tests. ** $p < 0.01$, *** $p < 0.001$, **** $p < 0.0001$. Note that all significant differences obtained by comparing NT titers **(a, b)** were replicated by the sVNT **(c, d)**.

Table S1. Cohort characteristics: Number of samples.

	Total (n)	Female (n)	Male (n)
**All subjects**	205	137	68
**Probable Omicron Primary Infection**	38	23	15
**Omicron Vaccine Breakthrough**	33	21	12
**2 x Vacc. (7-9 M)**	55	41	14
**3 x Vacc. (1 M)**	36	30	6
**Wildtype Infection**	43	20	23

Table S2. Cohort characteristics: Age (at blood sampling).

Cohort	Median (years)	Min	Max
**All subjects**	43.2	4.6	96.8
**Probable Omicron Primary Infection**	46.3	4.6	71.6
**Omicron Vaccine Breakthrough**	39.5	21.1	64.3
**2 x Vacc. (7-9 M)**	46.3	23.4	64.4
**3 x Vacc. (1 M)**	44.2	23.3	72.5
**Wildtype Infection**	33.6	16.5	96.8

Table S3. Summary of the vaccination status in Omicron Vaccine Breakthrough.

Vaccination 1	Vaccination 2	Vaccination 3	Number of samples
Pfizer	Pfizer	Pfizer	18
N/A	N/A	N/A	6
Pfizer	Pfizer	Moderna	4
Pfizer	Pfizer	N/A	2
Astra Zeneca	Astra Zeneca	Moderna	1
Moderna	Moderna	Moderna	1
Moderna	Moderna	-	1

N/A: Subjects reported e.g. "3 times vaccinated" bot no further details; "-" : only 2 times vaccinated Astra-Zeneca "Vaxzevria" ChAdOx1; Pfizer: Biontech/Pfizer "Comirnaty" *BNT162b2; Moderna: "Spikevax" mRNA-1273.*

Table S4. Summary of the timeline of infection, vaccination, and blood sampling.

<table>
<tr><th colspan="2">Cohort</th><th>Median</th><th>Range</th><th>Number of N/A</th></tr>
<tr><td>Omicron primary infection</td><td rowspan="3">Days between infection and blood sampling</td><td>41</td><td>16-107</td><td>0</td></tr>
<tr><td>WT infection</td><td>39</td><td>14-194</td><td>1</td></tr>
<tr><td rowspan="2">Omicron Vaccine Breakthrough</td><td>30</td><td>10-107</td><td>7</td></tr>
<tr><td>Days between the last vaccination and infection</td><td>140</td><td>48-372</td><td>4</td></tr>
<tr><td>2 x Vacc. (7-9 M)</td><td rowspan="2">Days between the last vaccination and blood sampling</td><td>251</td><td>212-286</td><td>3</td></tr>
<tr><td>3 x Vacc. (1 M)</td><td>30</td><td>15-70</td><td>1</td></tr>
</table>

Vacc.: Vaccinated; M: months post-vaccination; N/A: Subjects reported e.g., "3 times vaccinated" but no further details. Median and range were calculated excluding the missing data.

Table S5. Clinical characteristics of the cohort.

<table>
<tr><th>Cohort</th><th>Immune status</th><th>Comorbidities</th><th>Disease severity</th><th>Symptoms</th></tr>
<tr><td>2 x Vaccinated (7-9 M)</td><td rowspan="5">No report of immunosuppression / autoimmune disorder</td><td rowspan="5">No particular comorbidities reported</td><td rowspan="2">none</td><td rowspan="2">none</td></tr>
<tr><td>3 x Vaccinated (1 M)</td></tr>
<tr><td>Wildtype Infection</td><td rowspan="3">mild (no hospitalization or other medical intervention required)</td><td rowspan="3">fever, cough, headache, rhinitis, muscle aches, fatigue, sore throat (or asymptomatic detection in routine screenings)</td></tr>
<tr><td>Omicron Primary Infection</td></tr>
<tr><td>Omicron Vaccine Breakthrough</td></tr>
</table>

Table S6. Sensitivity and specificity of the sVNT to determine a NT titer ≥ 10 or to detect Omicron primary infection in different sub-cohorts.

	Cohort	n	Median NT titer	Median RBD Inh.	NT pos (n)	NT pos & sVNT pos (n)	NT neg (n)	NT neg & sVNT neg (n)	Sens. (%)	Spec. (%)
WT NT vs. WT RBD	2 x Vaccinated (7-9 M)	55	20	30	55	54	0	0	98 %	100%*
	3 x Vaccinated (1 M)	36	480	100	36	36	0	0	100%	100%*
	Wildtype Infection	43	30	16	43	39	0	0	90 %	100%*
	Omicron Primary Infection	38	<10	0	5	2	33	32	40%	97 %
	Omicron Vaccine Breakthrough	33	1280	100	33	33	0	0	100%	100%*
Omi-NT vs. Omi-RBD	2 x Vaccinated (7-9 M)	55	<10	0	2	12	40	43	17 %	93 %
	3 x Vaccinated (1 M)	36	160	60	32	36	0	0	89 %	100%*
	Wildtype Infection	43	<10	4	0	1	39	42	0 %	92 %
	Omicron Primary Infection	38	30	28	29	38	0	0	76 %	100%*
	Omicron Vaccine Breakthrough	33	480	100	32	33	0	0	97 %	100%*

Omi: Omicron; NT pos (n): number of samples with a NT titer >= 10; NT pos & sVNT pos (n): Number of samples with a NT titer >= 10 and sVNT result >= 7.5 (WT) or >= 18.5 (Omicron) (i.e. "true positive"). NT neg (n): number of samples with a NT tier < 10; NT neg & sVNT neg (n): number of samples with a NT titer < 10 and svNT tier < 7.5 (WT) or < 18.5 (Omicron). 100 %*: 0 NT negative samples in this cohort and 0 % were considered negative by the sVNT (i.e. theoretical specificity of 100%). Sens. (%): Sensitivity; Spec. (%): Specificity. Omi: Omicron; NT pos (n): number of samples with a NT titer >= 10; NT pos & sVNT pos (n): Number of samples with a NT titer >= 10 and sVNT result >= 7.5 (WT) or >= 18.5 (Omicron) (i.e. "true positive"). NT neg (n): number of samples with a NT tier < 10; NT neg & sVNT neg (n): number of samples with a NT titer < 10 and svNT tier < 7.5 (WT) or < 18.5 (Omicron). 100 %*: 0 NT negative samples in this cohort and 0 % were considered negative by the sVNT (i.e. theoretical specificity of 100%). Sens. (%): Sensitivity; Spec. (%): Specificity.
